# Stereotactic Ablative Brachytherapy: Recent Advances in Optimization of Radiobiological Cancer Therapy

**DOI:** 10.3390/cancers13143493

**Published:** 2021-07-12

**Authors:** Hui Xue, Bin Qiu, Hao Wang, Ping Jiang, Olga Sukocheva, Ruitai Fan, Lixiang Xue, Junjie Wang

**Affiliations:** 1 Department of Radiation Oncology, Peking University Third Hospital, Beijing 100191, China; xuehui@bjmu.edu.cn (H.X.); qiubin@pku.edu.cn (B.Q.); hhbysy@126.com (H.W.); bysyjiangping@163.com (P.J.); 2 Discipline of Health Sciences, College of Nursing and Health Sciences, Flinders University of South Australia, Bedford Park, SA 5042, Australia; olga.sukocheva@flinders.edu.au; 3 Department of Radiation Oncology, The First Affiliated Hospital of Zhengzhou University, Zhengzhou 450052, China; fanruitai@126.com

**Keywords:** brachytherapy, ablation, radiotherapy, cancer, seed implantation

## Abstract

**Simple Summary:**

Emerging studies involving ablative brachytherapy with curative effect have been published, but the evidence was not comprehensively discussed. This study will provide an overview of stereotactic ablative brachytherapy, focusing on the advances in stereotactic ablative brachytherapy optimization, and provide insights on the future benefits of the combined application of stereotactic ablative brachytherapy with cancer immunotherapies.

**Abstract:**

Brachytherapy (BT), a type of focal anti-cancer radiotherapy, delivers a highly focused radiation dose to localized tumors, sparing surrounding normal tissues. Recent technological advances have helped to increase the accuracy of BT and, thus, improve BT-based cancer treatment. Stereotactic ablative brachytherapy (SABT) was designed to improve the ablative effect of radiation, which was achieved via improved image guidance, and calculation of ablative dose, shorter treatment duration, and better organ preservation. Recently collected data characterized SABT as having the potential to cure various early-stage cancers. The method provides higher tumor control rate levels that were previously achievable only by surgical resection. Notably, SABT is suitable for application with unresectable malignancies. However, the pathological assessment of SABT irradiated tumors is limited due to difficulties in specimen acquisition. Prostate, lung, liver, and gynecological cancers are the most commonly reported SABT-treated malignancies. This study will give an overview of SABT, focusing on the advances in SABT optimization, and provide insights on the future benefits of the combined application of SABT with cancer immunotherapies.

## 1. Introduction

Radiotherapy (RT) has been successfully used for many decades and remains a standard cancer treatment option for many malignancies, with approximately half of all cancer patients worldwide receiving this type of therapy [1,2]. Tumor-targeting RT can be delivered using various methods, including external radiation (external beam radiotherapy, EBRT) or tissue/cavity-implanted radioactive (brachytherapy, BT) sources [2]. EBRT is the most frequently used method of RT in the world. The method has been technically improved and expanded during the past decades. It now includes stereotactic body radiotherapy (SBRT), intensity-modulated radiation therapy (IMRT), and hypofractionation regimen approach [2]. Accordingly, the novel RT treatment outcomes have been further improved, compared with conventional RT. One of the main advantages of the new RT modalities includes the possibility to eliminate small/early tumors. This early-stage treatment is achieved using stereotactic ablative radiotherapy (SABR), which is characterized by a high dose of radiation per fraction with a relatively short course of application [3].

BT, a specific type of RT, requires precise placement of radioactive source(s) directly into tumor tissues or next to it. As an anti-cancer treatment, BT has been used for more than 100 years [4]. The recent introductions of more precise image guidance, novel radioactive seeds (^125^I and ^103^Pd), BT treatment planning systems (BT-TPS), after-loading techniques and personalized three-dimensional printing templates (3D-PT) have dramatically improved the accuracy and BT clinical outcomes. Current technological advances allow strengthening the ablative effects in tumor tissues via focally delivered high radiation doses, which rapidly decline and minimize normal tissue damage [5,6,7]. Based on the calculation of radiation doses according to the inverse square law, stereotactic ablative BT (SABT) was designed for the exact delivery of radiation into malignant tissue. SABT protocol helps to avoid or minimize dose variations associated with tumor movement [8,9].

Considering that the SABT concept represents a relatively novel BT method, SABT-related clinical data was not comprehensively discussed. The method itself remains not well defined and requires critical analysis. Therefore, this review will discuss the clinical practice of SABT and analyze whether this method allows optimizing the operational efficiency and accuracy of cancer RT.

## 2. Main Principles of Ablative RT

The conventional RT dosage (i.e., 1.8–2 Gy/fraction; a total of 25–30 fractions [10]) and protocols are based on consideration of “4Rs” radiobiology principles [11]. The “4Rs” are the factors that directly define the outcome of the RT and include “repair of sublethal radiation-induced cellular/DNA damage”, “redistribution of cells within the cell cycle”, “reoxygenation of the surviving cells”, and “repopulation of cells after radiation” [11]. Intrinsic cell and tissue radiosensitivity were proposed as another important RT factor by Steel et al. in 1989 and is known as the 5th “R” which are important principles of conventional RT [8]. The recent development of highly conformal RT techniques allows following the 5R-principles closely, delivering high-dose radiation, and direct tumor ablation, while sparing the surrounding tissues and organs [9].

In 1951, Leksell et al. reported the utilization of gamma rays to focus radiation on intracranial targets and described the concept of stereotactic radiosurgery (SRS) [12]. SRS application is suitable for brain lesions and delivers the entire radiation dose in a single fraction [13]. However, organ movements represented a serious impediment and delayed the widespread introduction of stereotactic irradiation [14]. Therefore, the concept of SBRT was introduced and tested only in 2003 [15]. Currently, several common technical devices are used for SBRT, including non-coplanar/non-opposing arcs with incorporated conventional linear accelerator or robotic-based radiosurgery system. Nearly all cancer types may be treated using SBRT with 1–5 dose fractions. The observed SBRT clinical outcomes were comparable to those of surgical resection and physical ablation [16,17]. Both SRS and SBRT demonstrated advanced anti-tumor efficacy [18]. SABR approach, which combines both SBRT and SRS, was tested only a decade ago, in 2010, and indicated substantial treatment benefits regarding dose coverage of the cancer tissue volume [19]. Emerging clinical studies have confirmed that SABR not only directly ablates tumor cells but also triggers indirect anti-cancer effects, including stromal effects [20,21], vascular endothelial injury, and immune activation [22], which plays a crucial role in tumor elimination.

A great deal of progress has been made in the optimization of radiation dosimetry. However, there is still limited data available regarding the radiobiological effects of BT [23]. The 4Rs radiobiology fundamentals can be applied to BT by accounting for differences in dose rate, fractionation, and response to immunologic agents for this treatment modality. Dose rate is a major radiobiological parameter of BT, but few studies have evaluated other parameters involved in the differential effects of BT and little data is available regarding the impact of BT on tumor vascularization [24]. High dose rate-BT (HDR-BT) and pulsed dose rate modalities allow an optimization of dose distribution by varying the dwell times over the different dwell positions [25]. Low dose rate-BT (LDR-BT) has some radiobiological advantages compared to EBRT: sublethal damage repair during irradiation, leading to a relative protection of healthy tissue; no tumor cell repopulation; cell cycle redistribution; and a low oxygen enhancement ratio [25]. The effect of cancer cell repopulation during protracted irradiation is expected to be negligible for dose rates greater than 0.3 Gy/h [24]. The tumor microenvironment may also be involved in radiobiology of the regulation of BT effects. The unequalled, high dose gradient attained with BT may be optimal for enhancing the immunogenic response at the irradiated site while minimizing antagonistic effects on peripheral immune cells by avoiding irradiation of draining lymph nodes [23]. However, this aspect of radiobiology is poorly understood.

## 3. Characteristics of Modern BT

Accordingly, modern BT may be applied at either a HDR-BT or LDR-BT [26]. The dose conformity and accuracy of both HDR-BT and LDR-BT have been significantly improved with image guidance (ultrasound, computed tomography (CT), and magnetic resonance imaging (MRI), and BT-TPS [27]. Modern HDR-BT is employed via the assistance of computer-driven systems, with the guidance of electronic endoscope or ultrasound, which helps to control after-loading technique remotely. HDR-BT is mainly used for the treatment of solid cancers, including breast, skin, prostate, and cervical malignancies [28,29,30]. Image-guided radioactive seed implantation is used during LDR-BT [31]. New radioactive sources are now available for LDR-BT, including ^125^I (half live 59.6 days) and ^103^Pd (half-life 17 days). 3D-PT further improved the accuracy and post-plan dosimetry of the seed implantation [32]. LDR-BT was shown effective in the treatment of mostly reproductive system (endocrine) cancers, including prostate [33,34], uterine [35], cervical [36], and breast cancers [37].

### 3.1. Utilization of 3D After-Loading Machines

The old-fashioned HDR-BT was conducted using iridium wires which complicated the application due to imprecision of the source geometry. Moreover, the protocol was not safe for medical staff. Modern after-loading techniques use a single high-activity source, allowing a more precise radiation delivery and complete staff protection. During the procedure, an inactive catheter/applicator is introduced to the affected tissue/organ and then the radiation source is positioned through the catheter/container using a computer-driven remote-control system. Successful HDR-BT treatment has been reported in gynecological tumors (59% of all cases), prostate (17%), breast (9%), lung/bronchii (3%), and esophagus tumors (2%) [38].

### 3.2. 3D-PT and Increased Implantation Accuracy

**Figure 1:**
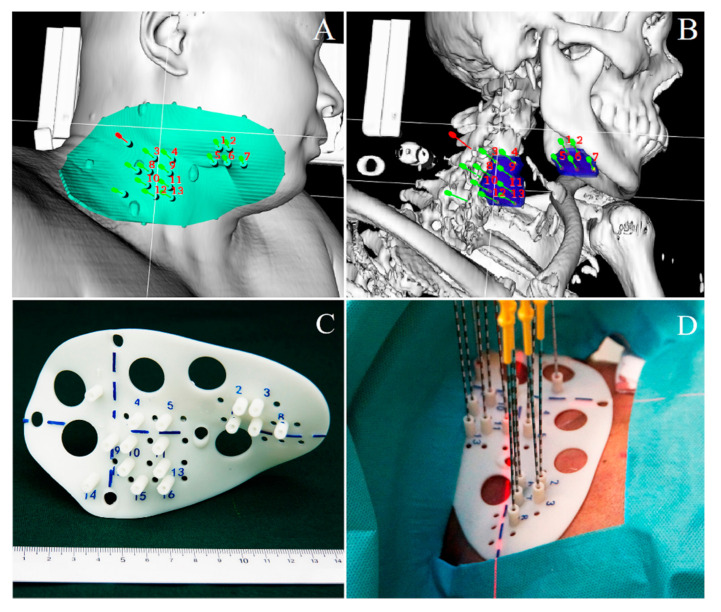
Three dimensional-printing templates (3D-PT) assisted individualized seed implantation. (**A**,**B**): Digital modeling of individualized 3D-PT in a patient with head and neck cancer; (**C**): The 3D-PT with 3 mm thickness contained information such as body-surface characteristics of the treatment area, localization markers, and an entrance hole for the 18-gauge needle; (**D**): 3D-PT was aligned to the therapeutic region, then needles were successfully inserted.

3D-PT was developed to assist computed tomography (CT)-guided seed implantation in tumors with prescribed radiation dose (usually biological equivalent dose (BED) >100 Gy) that required an accurate dose distribution (high dose conformity) [39,40]. The method can be also used to design an optimal dose for solid but moving tumors with irregular shapes. Before the introduction of 3D-PT-assisted seed implantation, the individual organ or target regions were simulated using a 3D-CT-based software package. The simulation allows the design of an optimal delivery and personalizes treatment. A calculated template with information about the planned optimal needle pathway, adequate position, and angulation of the insertion points are provided by software in 3D-printed form (Figure 1). The computerized approach facilitates the optimization of 3D-PT-assisted individualized seed implantation [7,39,40,41,42]. Guidelines, indications, and contradictions of optimal 3D-PT were described previously [43].

## 4. SABT Concept and Design

The concept of cancer tissue “ablation” was proposed by the Radiology Society of North America in 1997. The approach employs thermal energy to target fast-dividing tumor cells. The term “direct” was introduced to distinguish the method from intravenous, arterial, and oral routes. In contrast, to open surgery, ablation techniques are minimally invasive and involve physical energy (radiofrequency, microwave, cryoablation, ultrasound, and laser photothermal sources) to reach anti-cancer effectiveness [44].

**Figure 2:**
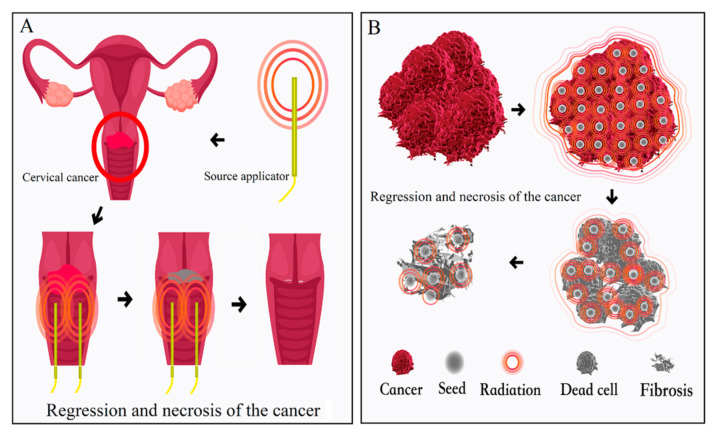
Stereotactic ablative brachytherapy (SABT) with high-prescribed radiation dose delivered to the tumor cell and tumor cell is directly ablated and replaced by fibrosis. (**A**): Intracavitary irradiation (**B**): Schematic process of cancer cell death after seed implantation.

SABT may be optimized to address specific cancer properties and locations that other ablation techniques may not be applicable to, including prostate cancer, cervical cancer, and head and neck cancers. SABT employs high doses of radiation for physical tumor eradication. The method has been used over the past 20 years and the term “SABT” was first proposed in-depth by Wang in Chinese in 2019 [45]. Numerous preclinical and clinical studies demonstrated SABT efficiency, especially in prostate and cervical cancer. A combination of after-loading and 3D-PT, as previously discussed in further detail, can facilitate the delivery of SABT in HDR-BT and LDR-BT, named HDR-SABT and LDR-SABT, respectively. High radiation doses were required, typically with about 80–90 Gy for HDR-SABT [30] and 110–160 Gy for LDR-SABT [39,46,47], which may cause complete tumor ablation, although further investigations are warranted to confirm the observed anti-cancer effects. The schematic diagram of the SABT-related effect is shown in Figure 2. The delivery of HDR-SABT is typically conducted with 4–8 Gy/fraction doses in a total of 3–6 fractions or using higher dose (>10 Gy per fraction) with only 1–2 fractions [30,48]. The delivery of LDR-SABT was administered mainly in the form of 3D-PT assisted seed implantation. Intraoperative implantation of a directional palladium sources was also reported and may be accurate for clinical use [49]. The success of SABT is associated with organ preservation and depends on several technological elements, including guided image acquisition, and correct calculations of dose and duration of treatment. Details of these technological advances are described below.

### 4.1. Image Guidance during Delivery of Treatment

To facilitate high dose optimization, consistency of the delivered treatment, and to broaden BT potential in cancer treatment, image guidance technologies are used during the design and delivery of HDR-BT and LDR-BT [50]. Ultrasound guidance is employed in BT during prostate and breast cancer treatments [51,52]. Computed tomography (CT) guidance is mainly used for seed implantation into cancers located at head and neck, thoracic, primary/metastatic liver/adrenal, recurrent/metastatic pelvic cavity, and spinal areas [29,33,43]. Recent testing of electromagnetic tracking of endoscopes and BT applicators indicated higher precision of HDR-BT positioning in cases with complex insertions [53]. MRI guidance is less accessible in the clinic than ultrasound and/or CT guidance, although is regularly used during pre-BT assessments [54]. However, MRI assessment is considered to be superior to CT scans of soft tissue tumors, because MRI allows accurately define precise tumor shape and location [55].

### 4.2. Precise Calculation of RT Dose/Duration Defines HDR and LDR Effectiveness and Curative Effects

Precise calculation of RT dose in-tissues distribution nearby the BT sources is essential for optimized anti-cancer treatment, considering tissue heterogeneity. Cancer-related parameters are calculated using dose-volume histogram and CT-based TPS [56]. High dose optimization and conformity of ablative BT allow achieving advanced curative effects. For instance, HDR-BT was shown effective in early-stage intra-tracheal [53] and cervical cancers [57], while LDR-BT is successfully used in prostate cancers [58]. Recent retrospective analysis indicated that HDR-BT patients had a lower incidence of acute genitourinary toxicities (grade ≥ 2) [59]. The same study suggested that HDR-BT in combination with external beam RT may serve as a good alternative to LDR-BT (±EBRT) [59]. The precisely calculated ablative dose is the major advantage that facilitates the achievement of better curative effects using SABT, compared to palliative BT. However, the pathological (immunohistochemical (IHC)) assessment of SABT-irradiated tumors is limited due to technical difficulties in acquiring specimens. Accordingly, SABT curative effects are largely assessed using observed improvements in tumor control rate. Conclusively, multiple studies have confirmed that BT irradiation methods delivered advanced curative outcomes and did not result in severe/acute gastrointestinal adverse effects [51,55,58]. There are novel methods, including urethral D10% color map (dose-area evaluation method), that help to reduce BT-related toxicity [60].

### 4.3. SABT Allows Shortening the Duration of the RT Exposure

Conventional EBRT is usually repeated 5 times a week using 1.80–2.0 Gy per fraction. EBRT is generally completed in 5–7 weeks. For hypo-fractionated curative treatment, the regimens are usually delivered within 10 fractions for 2–3 weeks [61]. Compared to conventional EBRT, modern HDR- and LDR-BT are delivered in relatively short times. Afterloading HDR-BT is hypo-fractionated and typically delivered in 1–10 fractions of 3–20 Gy depending on the indication [62]. Accordingly, LDR-BT seed implantation is a one-time procedure that provides a short duration but effective treatment [63]. However, shortened duration of treatment is meaningless when BT is used with inoperable tumors because the seed implantation is usually repeated to achieve tumor eradication [64,65].

### 4.4. BT Facilitates Organ Preservation

Significant advantages in the preservation of tissue/organ functions were observed in patients after SABT compared to surgical resection/EBRT. During SABT, the radiation dose to surrounding tissues rapidly decreases which allows preservation surrounding normal tissues. HDR-BT is the minimally invasive treatment used in lung or cervical cancers with the after-loading technique. Seed implantation, although considered an invasive procedure, permits preservation of organ functions which was observed during other interventional ablative techniques, including cryotherapy, radiofrequency ablation, and microwave ablation [4]. Good survival outcomes and a minimal complication rate, compared to surgical resections, were observed in organ-preserving BT of the bladder [66], rectal [67], and prostate [68] cancers.

## 5. The Practice of SABT: Outcomes, Advances, and Perspectives

Using the search keywords “ablative” and “radiotherapy” on PubMed, we retrieved published studies (1 April 1972, to 31 March 2021) involving ablative BT with the relevant information about the local controls, failure rates, and survival parameters that are listed in Table 1. According to the collection and reviewed studies, it has been determined that SABT is a suitable anti-cancer cure that was tested in various early-stage cancers, including prostate (most reported) [69,70], lung [5], liver [71], and cervical [72] cancers. However, SABT is rarely used for the treatment of other cancer types. The most relevant key studies that used SABT are reviewed and discussed in the following sections below.

### 5.1. Application of SABT in Prostate Cancer Patients

Successful application of BT has been shown in numerous studies with prostate cancers. Biochemically marked recurrence was rarely reported but was more frequently observed in patients with intermediate-risk cancers and/or in older patients [70]. However, a meta-analysis [73], which included a large data set (26,129) from BT-treated patients in 41 studies (two randomized controlled trials (RCTs) and 39 Non-RCTs), provided no significant evidence that SABT is inferior to standard EBRT. Considering the treatment outcomes in a 5-year follow-up, the rate of biochemical failure was lower in SABT-treated patients compared to those after EBRT [74].

According to the Genitourinary Radiation Oncologists of Canada Prostate Cancer Risk Stratification database, low-risk prostate cancer patients receiving LDR-BT had a failure-free survival rate similar to SABR-treated patients [69]. Radioactive seed implantation is the optimal treatment for early-stage prostate cancer, with a recommended prescription dose of 140–160 Gy [43,47]. Focal ablative dose-escalated radiation is also feasible using the proposed protocol with MRI-guided HDR-BT [83]. Kovács et al. [84] reported that 130 patients with localized prostate cancers treated with EBRT (50 Gy followed by 30 Gy) in 2 fractions of HDR-BT boost had excellent long-term outcome data (93% of the patients with prostate-specific antigen nadir ≤1 ng/mL during a 4.3 year median follow-up) and no enhancement in late treatment toxicity.

However, there are limited publications with analysis of the long-term effects of SABT. It remains unclear whether SABT helps to achieve lower morbidity rates during longer (>5 years) periods compared to standard EBRTs. BT and radical prostatectomy were comparable in terms of quality of life and biochemical progression-free survival while favoring BT regarding patient satisfaction and sexual function [85]. However, the quality of life in patients with advanced malignancies may be improved with I-seed^125^ implantation using a 3D-printed personalized template/CT guidance [86]. The study used the European Organization for Research on Treatment of Cancer Quality of Life Questionnaire-C30, which has certain limitations [86]. Further comparative studies are warranted to address SABT efficacy, quality of life, and economic outcomes using a broader variety of cancer-risk patients and longer follow-up terms [69].

### 5.2. Application of SABT in Lung Cancer Patients

BT is a promising approach in treating lung cancers with a recommended prescription dose range of 100–125 Gy. The dosage distribution has been designed in consideration of the normal tissue dose limitation and preservation of critical thorax-located organs (lung, bronchus, nerves, and spine cord). Accordingly, the doses rapidly decline outside tumors [29,87]. Several studies reported that SABT improved local cancer control and prolonged the survival of certain patients with lung cancer [29,88].

HDR-SABT (endobronchial BT) has been effective in patients with early-stage lung cancer confined to the endobronchial lumen. Soror et al. [89] observed 126 patients with endobronchial tumor recurrence treated with HDR endobronchial BT. Surgery and external beam radiotherapy were contraindicated in these patients. The study reported an 86.5% rate for 3-month complete local response, 41.4% disease-free survival rate, and 23.6% overall survival at 5 years, with 12.7% of patient mortality caused by massive hemoptysis. The effect was observed in numerous clinical studies with promising outcomes and complete cures in selected patients [29]. The SABT lung malignancy control rate is > 80%, while the tracheal obstruction remission rate is about 60–80% [90,91,92]. The largest retrospective study by Aumont-le Guilcher et al. [90] reported data of 266 patients with endotracheal early lung cancers that were treated with endobronchial BT. The overall BT response rate at 3 months was 93.6%, while the 2- and 5-year survival rates were 57% and 29%, respectively [90].

In early-stage lung cancers that are not suitable for surgical resection or EBRT, LDR-SABT could be used as an alternative anti-cancer treatment with interstitial radioactive seed implantation and image guidance [27,29]. The prescription dose of radioactive seed was generally 100–120 Gy in lung cancer studies [6,42,77,93,94,95,96,97,98,99,100]. Another study reported that the local cancer control rate was 80–100% with promising 1- and 2-year survival rates (90–95% and 70–80%, respectively) for early-stage lung cancer [27]. The efficacy of template-assisted SABT after neoadjuvant therapy was also declared to be significant in patients with inoperable peripheral lung cancer [6]. Furthermore, in patients with locally advanced (stage III) non-small cell lung cancer, the combination of CT-guided BT and bronchial arterial chemoembolization was found efficient and safe even after the failure of concurrent chemoradiotherapy [63]. However, clinical BT advantages and clinical lung cancer outcomes were reported by very few randomized trials [101,102,103]. Therefore, larger prospective studies are urgently needed to confirm the observed data.

### 5.3. Application of SABT for Treatment of Gynecological Tumors

It was estimated that the overall cure rate for cervical cancer in the United States may be improved using high-quality BT in selected patients [104]. The observed effects were also associated with a moderate rate of treatment-related morbidity [72]. Currently, the hybrid inverse planning optimization method in cervical BT delivered reasonable plans, although further testing is required to boost the technique [105].

Combined with chemotherapy and EBRT, HDR-BT was recommended for the nonsurgical management of stage I to III cervical cancers [104]. Accordingly, BT anti-cancer enhancement has been associated with significantly improved outcomes [106]. Concurrent chemo-radiotherapy plus image-guided adaptive intracavitary BT in advanced malignancies resulted in local control rates of 95–100% (at 3 years) in limited/favorable (IB/IIB) and 85–90% in large/poor response (IIB/III/IV) cervical cancer patients [72]. Using the National Cancer Data Base, Gill et al. [107] analyzed the anti-cancer effects of the radiation dose-escalation technique in 7654 patients with cervical cancer. The median survival time of BT-treated patients was 70.9 months that was significantly higher compared with the survival period (47.1 months) of those patients who were treated with either IMRT or SBRT [107]. Furthermore, a couple of systematic reviews [108,109] reported low toxicity rates after HDR-BT in Grade 3–4 genitourinary (0–12%) and gastrointestinal (0–8%) cancers in phase I/II studies (≥4-year median follow-up time).

Interstitial CT-guided seed implantation was found as harmless and practical in patients with recurrent ovarian cancer who failed to respond to a variety of anti-cancer therapie [110]. The study data indicated very limited complications. Notably, BT delivered dramatic pain relief (61.5%) and amended the general living quality of those patients [110]. However, larger clinical studies are required to confirm these findings.

### 5.4. Application of SABT in Liver Cancer Patients

Several decades ago, radiation therapy was considered unsafe for application in liver cancer patients. The conclusion was associated with liver cell intolerance to high radiation doses [111]. Consequently, BT has been rarely used for the treatment of hepatocellular carcinomas, unless the salvage conditions influenced the therapy choice. During the last decade, ablative radiotherapy has been revolutionized. BT approach in patients with liver tumors had been modernized and ablative BT was increasingly used as a non-thermal ablation [111,112].

In patients with liver cancer, BT application allows achieving an excellent local tumor control of up to 96.1% [112]. Moreover, BT delivered higher survival benefit compared to the best supportive care (median overall survival 23 months vs. 5 months) in those patients [112]. Subir et al. [71] reported on 64 patients with unresectable or residual disease after surgical resection for intrahepatic malignancies who underwent 160 Gy permanent Iodine-125 BT. The median length of follow-up in the study was 13.2 years. The overall 1-, 3-, and 5-year actuarial intrahepatic local control rates were 44%, 22%, and 22%, respectively. The 1-, 3-, and 5-year actuarial overall survival rates were 73%, 23%, and 5%, respectively. The authors concluded that, for select patients with unresectable primary and metastatic liver tumors, SABT is a safe and effective alternative to other locally ablative techniques [71]. Moreover, SABT can provide long-term local control and increased survival in metastatic liver cancer [71]. Pennington et al. [113] reported SABT-based treatment of liver metastasis using a higher cancer-targeting dose with a similar dose to organs at risk, but potentially lower target coverage compared with SABR (five 12 Gy fractions). 

Furthermore, for secondary liver cancers/metastases, BT demonstrated promising local tumor control rates of 74.9–97.4%, depending on the primary site, including 74.9–87.1% in colorectal cancer, 96.5–97.4% in breast cancer, and 90% in pancreatic cancer [112]. Another recent report about 194 patients with unresectable liver metastases, confirmed that interstitial BT proved an effective cure for small and large liver metastases from rare or less common cancers [114]. Current guidelines for BT in liver cancer patients are being revise [115]. However, further larger investigations are warranted.

### 5.5. SABT in Other Cancers 

BT application was tested in different unresectable tumors. For instance, Ruge et al. [75] utilized SABT for the treatment of singular brain metastases (90 cases). The study demonstrated a remarkable 1-year local cerebral relapse rate of only 5.4% [75]. In another similar study that compared SABT (77 cases) with SRS (142 cases), the 1-year local cancer control rate with SABT was 96.7% compared to the 93.6% rate with SRS (no significant differences) [76]. Because SABT allows histological (re-)evaluation and treatment within 1 stereotactic operation, the procedure is less restricted by tumor localization or size and, therefore, greatly advances local treatment options. 

**Figure 3:**
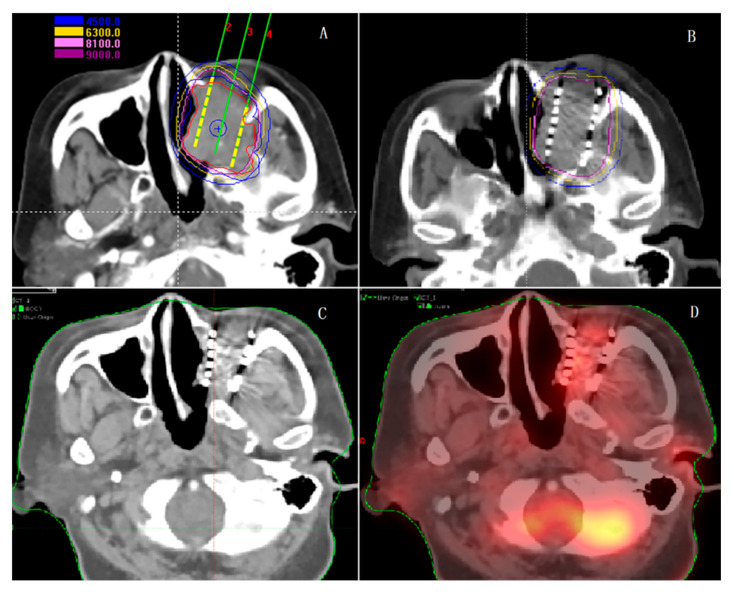
Clinical stereotactic ablative brachytherapy (SABT) practice for head and neck cancers. (**A**): Preoperative planning for a head and neck cancer; (**B**): Intraoperative replan and seed implantation; (**C**,**D**): the cancer is shrinking 3 months after seed implantation with low in-taking of fluorinated deoxy-glucose on the PET/CT image (SUV < 2.5).

Notably, SABT does not preclude the possibility of additional radiation treatment in the event of disease relapse. Accordingly, accelerated partial breast irradiation is an attractive adjuvant approach in selected patients with breast cancer. BT is performed as perioperative or postoperative breast cancer treatment and consists of placing sources within the tumor bed to decrease the risk of local relapse and provide a better dosimetry profile to the skin [4,116]. MRI-guided single fraction radiotherapy with an integrated ablative boost to the tumor is dosimetrically feasible with an interstitial multi-catheter BT [117]. A decrease in long-term morbidity was also reported in some other clinical situations, such as head and neck cancers [32], anal/colorectal cancers [118], esophageal cancer [119], gynecological carcinomas [120], and penile cancer [4]; although SABT-based clinical testing remains under-addressed. Figure 3 demonstrates clinical SABT practice for head and neck cancers.

## 6. Future Perspectives

Advances in SABT-associated techniques, including immune activation, needle/applicator navigation guidance, and 3D-PT assistance, are considered novel concepts that deserve clinical evaluation and proper assessment. For instance, RT/BT was indicated as a potential partner for cancer-targeting immunotherapies and a method of in situ tumor vaccination via increase immunogenicity [121]. RT/BT was shown to boost immunotherapy via increased local inflammation, modulation of suppressive lymphocyte lineages, and cancer cell sensitization to immunogenic cell death [122]. However, a limited of supportive clinical evidence and unclear potential benefits that currently exist do not persuade clinicians to test this technique. Combined SABT and EBRT are not considered by many clinicians, although the combination represents an advanced opportunity in cancer eradication. Promising SABT clinical outcomes in advanced unrespectable malignancies inspired the initiation of several clinical trials that aim to investigate the benefits of BT in combination with immunotherapies [123,124]. Future trial data should clarify the benefits of BT in resistant and unresectable cancers.

## 7. Conclusions

Although SABT is not a novel technique in clinical anti-cancer practice, its optimal capacity has not been sufficiently explored. Considering unresectable tumors, a need for more extensive BT evaluation is warranted.

## Figures and Tables

**Table 1:** Published literature involving ablative brachytherapy with reported local control/failure rate/survival.

Author(s) (Reference)	Design	Year	Cases	Mean/Median Age (y)	Male (%)	Cancer	Treatment	Outcomes
Nag et al. [71]	Retrospective study	2006	64	57.4	31	Intrahepatic malignancies	160-Gy permanent I brachytherapy	1-y, 3-ys, 5-ys LCR 44%, 22%, and 22%; 1-y, 3-ys, 5-ys OS rate 73%, 23%, and 5%
Ruge et al. [75]	Retrospective study	2011	90	59	48	Brain Metastases	SBT	1-y local cerebral relapse 5.4%
Ruge et al. [76]	Retrospective study	2011	142SRS/77SBT	58/58	82/35	Cerebral Metastases	SRS vs. SBT	1-y LCR SRS/SBT 93.6%vs.96.7%
Pötter et al. [72]	Prospective study	2011	156	58	0	Cervix cancer (FIGO stages IB–IVA)	EBRT ± chemotherapy + HDR brachytherapy	Complete remission 97%; 3-ys LCR 95%; 3-ys survival 68%
Tselis et al. [77]	Retrospective study	2011	55	64	37	Metastatic/primary intrathoracic malignancies	HDR brachytherapy	1-y, 2-ys, 3-ys LCR 93%, 82% and 82% for metastatic/86%, 79%, and 73% for primary cancer
Hoskin et al. [78]	Phase II study	2017	293	69	293	Prostate cancer	HDR brachytherapy	4-ys bPFS 91%-94%
Loblaw et al. [69]	Propensity score matching	2017	71SABR/213LDR	64.93	284	Low risk localised prostate cancer	SABR/LDR	6-ys biochemical failure-free survival SABR 97.1% versus LDR 93.4%
Taussky et al. [70]	Retrospective study	2018	454	66	454	Low- or intermediate-risk prostate cancer	LDR prostate brachytherapy	7-ys recurrence-free survival 96%
Mulherkar et al. [79]	Propensity-matched study	2019	52Radiation/419surgery	69	471	Early-stage penile cancer	Brachytherapy/EBRT/surgery	5-ys OS: definitive radiation vs. surgery 61.6% vs. 62.2%
Damm et al. [80]	Prospective study	2019	16	76	11	Renal masses	HDR brachytherapy	LCR 95% (median follow-up 22.5 months)
Pang et al. [5]	Clinical trial	2019	33	55	13	Peripheral lung cancer (stage I 4, II 14, and III 15)	Ir source stereotactic ablative brachytherapy	CR plus PR at 6-month 100%
Tharmalingam et al. [81]	Multicenter prospective	2020	441	73	441	Prostate cancer	HDR brachytherapy	2-ys bPFS 94% and 3-ys bPFS 88%
Langley et al. [82]	Phase II prospective trial	2020	30	65.6	30	Low or intermediate-risk unilateral localised prostate cancer	Hemi-Ablative LDR brachytherapy	PSA was reduced at 24 months by 78%

LCR = Local control rate; OS = Overall survival; y = year; ys = years; SRS = Stereotactic radiosurgery; SBT = Stereotactic brachytherapy; LDR = Low-Dose-Rate; HDR = High-Dose-Rate; FIGO = International Federation of Gynecology and Obstetrics; EBRT = External beam radiotherapy; SABR = Stereotactic ablation radiotherapy; bPFS = biochemical progression-free survival; CR = Complete response; PR = Partial response; I = iodine125; Ir = Iridium192.
